# Factor Structure and Measurement Invariance Across Gender Groups of the 15-Item Geriatric Depression Scale Among Chinese Elders

**DOI:** 10.3389/fpsyg.2019.01360

**Published:** 2019-06-21

**Authors:** Haofei Zhao, Jiayue He, Jinyao Yi, Shuqiao Yao

**Affiliations:** Medical Psychological Center, Second Xiangya Hospital, Central South University, Changsha, China

**Keywords:** depression, factor structure, measurement invariance, Chinese elders, gender differences

## Abstract

The 15-item Geriatric Depression Scale (GDS-15) is widely used to screen depression among elders. But the factor structure of the Chinese version GDS-15 remains unclear. This study was conducted to determine the best-fit factor structure of GDS-15 and to assess measurement invariance across gender groups in a sample of Chinese elders recruited from Mainland China (final sample *N* = 2428). The best-fit factor structure was examined by confirmatory factor analysis (CFA). Multigroup CFA was utilized to test the measurement invariance across genders of the factor structure. The results of CFA revealed that a three-factor model, including life satisfaction (four items), general depressive affect (seven items), and withdrawal (three items), fits the structure of the GDS-15 best. Measurement invariance across genders was supported, fully assuming different degrees of invariance.

## Introduction

Depression is a common mental disorder among older adults, with some 15% of community-dwelling older adults experiencing clinically significant depressive symptoms (Blazer, 2003). Late-life depression is linked to serious consequences, such as impaired daily functioning, increased health care use, and reduced quality of life (Castelo et al., 2010). Hence, assessment of depressive symptoms is an important mental health evaluation in this population.

The Geriatric Depression Scale (GDS), which was the first screening instrument to be tailored to geriatric patients (Yesavage et al., 1982), has become widely used to measure depression levels in the elderly. To reduce the time required for GDS administration and thus avoid respondent fatigue, a 15-item short-form GDS was developed from the original 30-item scale (Sheik and Yesavage, 1986). Unlike other depression tools such as the Epidemiological Studies Depression Scale (CES-D) and the Beck Depression Inventory (BDI), both versions of the GDS do not contain somatic items that may be less valid because they are common in elders (Sheik and Yesavage, 1986; Stiles and Mcgarrahan, 1998). Moreover, items of GDS use an easy response format (yes/no) preferred among older respondents. The 15-item GDS (GDS-15) retains the advantages of the original 30-item GDS, including simplicity of administration, an easy response format, and economy of time, and its validity and reliability have been demonstrated repeatedly (Cwikel and Ritchie, 1989; Lesher and Berryhill, 1994; Almeida and Almeida, 1999; Fountoulakis et al., 1999; Tang et al., 2005; Chaaya et al., 2008). Both ICD-10 criteria and DSM-IV criteria have shown that the GDS-15 is valid for measuring depression (Almeida and Almeida, 1999). GDS-15 may have more practical appeal because of the time restraints faced in clinical practice (Yao et al., 2009). In addition, the scale has been translated into multiple languages and translated versions have been proved for assessing depressive symptoms in people from various ethnic backgrounds (Iwamasa et al., 1998; Liu et al., 1998; Ishine et al., 2005; Malakouti et al., 2006; Onishi et al., 2006; Chiesi et al., 2018), including ethnic Chinese people living in Western countries (Mui, 1996; Lai, 2000).

Although the psychometric properties of the long and short GDS scales have been documented (Jang et al., 2001; Broekman et al., 2008; Pocklington et al., 2016), the factor structure of the Chinese version GDS-15 is still unclear. Mitchell et al. (1993) first proposed a three-factor model: general depressive affect (seven items), life satisfaction (four items), and withdrawal (three items). Item 10 “memory” failed to fit any of these factors. However, a number of other studies have reported different GDS-15 structures with two (Mui, 1996; Friedman et al., 2005; Brown et al., 2007), three (Incalzi et al., 2003; Imai et al., 2014), and four (Onishi et al., 2004; Lai et al., 2010) factors. Results of previous studies investigating the factor structure of the Chinese version GDS-15 have been mixed. Mui (1996) reported a two-factor model consisting of “happy mood” and “sad mood.” Implementing the GDS-15 among aging Chinese in Canada, Lai and Colleagues reported a two-factor model (i.e., affective mood, cognitive mood; Lai, 2000) and a more detailed four-factor model (i.e., positive mood, negative mood, inferiority/disinterested, uncertainty, Lai et al., 2005). Most subjects of the studies above lived in Western societies. Only one study employing exploratory factor analysis (EFA) and confirmatory factor analysis (CFA) reported a four-factor solution focused on depression among aging Chinese in Mainland China, with the following factors: positive and negative mood, energy level, inferiority, and disinterested (Lai et al., 2010). Researchers have deduced that the differences of these factor models may be related to cultural differences in the concept and expression of depression (Kim et al., 2013). For example, dominant social values of people in Western countries are individualism and personal level democratic values, whereas Chinese living in Mainland China takes more value on collectivism and at-large benefits, due to a different political and social system. These differences above in beliefs and social contexts play an important role in personal expression of affection (Mui, 2010; Kim et al., 2013).

Findings obtained depending on samples from Western societies may not necessarily be applicable to the older adults in Mainland China. The study of Lai et al. (2010) focused only on lonely elder Chinese. It is necessary for us to examine which factor structure model is more suitable for Chinese elders, for which will be helpful for developing a standardized scoring method and enable us to explore any differences across studies. In the current study, CFA was conducted to compare factor structure models that were identified in previous studies. GDS-15 total score is usually used in practice and research. However, a total score should not be used unless the covariance between the first-order factors is adequately explained by the second-order factor (Marsh and Hocevar, 1985). There are no published studies of the second-order factor of GDS-15 reported; thus, we performed a second-order factor analysis to confirm the validity of GDS-15 total scores. The trend of women having more depression problems than men was recapitulated (Nolen-Hoeksema, 2001). Tang et al. (2005) have examined the differential item functioning (DIF) of GDS-15 items, but the study was based on a sample of Hong Kong Chinese patients with pneumoconiosis. No study has tested the measurement invariance of the GDS-15 across genders in the mainland Chinese population. As related to gender, if the measurement invariance does not hold across groups, differences in observed scores may not be directly comparable (Wang et al., 2013). The true differences across groups may be mixed with the measurement bias of assessment. Exploring measurement invariance is beneficial for increasing the accuracy of depression assessments and the comparability across groups.

Hence, to develop the Chinese version of GDS-15, the first purpose of this study was to examine the best factor structure of GDS-15 in a large representative sample. A second purpose was to test the gender invariance of the GDS-15. We employed the CFA to compare the existing factor models from previous studies. Second-order CFA was performed to confirm the validity of the GDS-15 total score. Subsequently, we assessed the measurement invariance across genders of the best-fitting model.

## Materials and Methods

### Sample

The inclusion criteria were as follows: age of 60–99 years old and ethnic Chinese resident of Beijing, Hunan, and Shandong province, China. The exclusion criteria were as follows: diagnosed with severe mental illness; insufficient cognitive ability to understand the questionnaire; unable to understand Mandarin and therefore unable to complete the questionnaire; cannot fill out the questionnaire due to other reasons. This study investigated the level of depression in the elderly, with 2,470 participants, and 42 failed to respond to all GDS-15 items. The final sample of 2,428 elderly Chinese volunteers included 1,141 men (47.0%) and 1,287 women (53.0%). The mean age of the men was 73.14 years [standard deviation (*SD*) = 8.07], and the mean age of the women was 71.78 years (*SD* = 7.70).

### Study Design

Postgraduate psychology researchers in China were recruited and trained to do this work. Participants completed the survey in a district activity center and elderly with visual impairment or lack of formal education would get support from researchers. The study was approved by the Ethics Committee of the Second Xiangya Hospital of Central South University. Each participant gave written informed consent prior to their inclusion in the study.

### Depression Symptom Assessment

The Chinese version of the GDS-15, wherein each item was a yes or no question, was used to measure depressive symptoms. The positive depression symptom response was yes for 10 items and no for 5 items, such that a point was marked for each positive symptom response. Thus, higher values indicated more depressive symptoms. As recommended by a study conducted among Chinese elders (Boey, 2000), we adopted 8 as the cutoff score. Both validity and reliability of the GDS-15 were validated satisfactory among Chinese elders in previous studies (Mui, 1996; Liu and Guo, 2008). In the current study, the scale has been confirmed to show good internal consistency (Cronbach’s α = 0.873).

### Statistical Analyses

Preliminary analyses were done in SPSS Version 22 (IBM, 2013), and CFA was conducted in Mplus7.4 (Muthén and Muthén, 1998). Given that the response options of items were binary (yes and no), the maximum-likelihood (ML) estimator is not adequate as it could bias the results. The robust weighted least squares with mean and variance adjustment (WLSMV) estimator was used, which could account for the binary response scaling (Finney and DiStefano, 2013; Morin et al., 2017). The whole sample was randomly divided into sample 1 (*n* = 1,174) and sample 2 (*n* = 1,254). This method of randomly assigning a larger sample into two independent samples is a common approach (Lai et al., 2010; Wang et al., 2012; He et al., 2018).

We employed CFA in sample 1 to compare competing models and determine the best-fitting factor model. A total of seven competing models were compared (Table 1). Models from different versions of GDS-15 were not included in the current analysis. Regular chi-square difference tests were not conducted here for the comparison of non-nested competing models. Following generally accepted practice, we used the Tucker–Lewis index (TLI), the chi-square, comparative fit index (CFI), and root mean square error of approximation (RMSEA) to evaluate the fit of each model. CFI and TLI values ≥0.90 indicate adequate model fit (0.95, excellent fit), while RMSEA values ≤0.08 and 0.06 indicate acceptable and excellent, respectively (Kline, 2010; Vrieze, 2012).

**Table 1:** Characteristics of each factor model tested.

Model	Method	Number of factors	Factor 1 items	Factor 2 items	Factor 3 items	Factor 4 items
Mitchell et al., 1993	EFA	3	3, 4, 6, 8, 12, 14, 15	1, 5, 7, 11	2, 9, 13	—
Incalzi et al., 2003	EFA	3	1, 5, 7, 11	3, 4, 8, 10, 14	2, 12, 13, 15	—
Onishi et al., 2004	EFA	4	1, 5, 7, 11, 15	2, 3, 4, 6, 8	12, 13, 14	9, 10
Friedman et al., 2005	EFA	2	2, 3, 4, 6, 8, 9, 10, 12, 13, 14, 15	1, 5, 7, 11	—	—
Brown et al., 2007	EFA	2	2, 3, 4, 6, 8, 9, 10, 12, 14, 15	1, 5, 7, 11, 13	—	—
Lai et al., 2010	EFA + CFA	4	1, 3, 4, 6, 7, 11	5, 8, 13	12, 14, 15	2, 9, 10
Imai et al., 2014	EFA + CFA	3	2, 6, 8, 9, 10, 13, 14, 15	1, 5, 7, 11	3, 4, 12	—


*EFA, exploratory factor analysis; CFA, confirmatory factor analysis.*

We hypothesize that there is a higher-order factor Geriatric Depression that accounts for the commonality among first-order factors. First-order CFA was conducted in sample 2 to validate the best-fitting structure of the GDS-15 confirmed in sample 1. Subsequently, second-order CFA was performed to calculate the target coefficient that could be used to decide whether the first-order factors were adequately explained by the higher-order factor. As recommended by Comrey and Lee (2013), the magnitude of the factor loadings was interpreted as follows: ≥0.71, excellent; 0.63–0.70, very good; 0.55–0.62, good; 0.33–0.44, fair; ≤0.32, poor.

Multigroup CFA was implemented in the whole sample to test gender invariance of the best-fitting model. We considered four aspects of invariance including configural invariance (Model A), metric invariance (Model B), scalar invariance (Model C), and strict invariance (Model D). Model A was used to evaluate the structure of latent variables, and the results of which served as a baseline model. Model B was tested based on the results of configural invariance with factor loading equivalence constraints imposed to ensure similarity of the observed indicators and underlying traits across gender. Model C was based on the result of the last step and in which we constrained variable intercepts equal. Model D test was conducted with factor loadings, variable intercepts, and error variance constraints equally set. As suggested by Cheung and Rensvold (2002), CFI, TLI, and RMSEA changes were employed to evaluate invariance; ΔCFI ≤0.01, ΔTLL ≤0.01, and ΔRMSEA ≤0.015 were considered evidence of invariance (Cheung and Rensvold, 2002; Chen, 2007).

## Results

### Preliminary Analyses

In the whole sample, the GDS-15 total scores had a mean (SD) of 4.03 ± 3.88 for males and 4.59 ± 4.10 for females. The GDS-15 total scores range was 0–15, with women having significantly higher scores than men (*t* = 3.46, *df* = 2,426, *p* < 0.05). Mean (SD) GDS-15 total scores did not differ significantly (*t* = 0.46, *df* = 2,426, *p* > 0.05) between sample 1 (4.29 ± 3.96) and sample 2 (4.36 ± 4.04). When score ≥8 was used as the cutoff score, 19.9% of the participant showed significant depressive symptoms.

### Factor Structure of GDS-15

As reported in Table 2, we obtained good fit indexes in all examined models. CFIs, TLIs, and RMSEAs were >0.95, >0.95, and <0.08, respectively. The best-fitting model was Mitchell’s three-factor model (WLSMV χ^2^ = 260.316, *df* = 74, TLI = 0.989, CFI = 0.991, RMSEA = 0.046). Next was Brown’s two-factor model (WLSMV χ^2^ = 438.968, *df* = 89, TLI = 0.980, CFI = 0.983, RMSEA = 0.058). For item 10 in Brown’s model, the factor loading loaded on its latent factor was 0.116 (<0.32), a poor loading. Therefore, the best-fitting model for older Chinese was Mitchell’s three-factor model. The results of first-order CFA in sample 2 showed that the three-factor model had an excellent fit to the data (Table 2). The correlations between the three factors in sample 1 ranged from 0.823 to 0.955 and those between the three factors in sample 2 ranged from 0.878 to 0.950 (see Table 3). All correlation coefficients were positive and statistically significant (*p* < 0.001).

**Table 2:** Goodness-of-fit indices of the compared models.

Model	WLSMV χ^2^	*df*	*p*	TLI	CFI	RMSEA (90% CI)
Mitchell et al., 1993	260.316	74	<0.01	0.989	0.991	0.046 (0.040, 0.052)
Incalzi et al., 2003	390.318	62	<0.01	0.980	0.984	0.067 (0.061, 0.074)
Onishi et al., 2004	461.223	84	<0.01	0.977	0.982	0.062 (0.056, 0.067)
Friedman et al., 2005	464.564	84	<0.01	0.979	0.982	0.060 (0.055, 0.065)
Brown et al., 2007	438.968	89	<0.01	0.980	0.983	0.058 (0.053, 0.063)
Lai et al., 2010	454.825	84	<0.01	0.978	0.982	0.061 (0.056, 0.067)
Imai et al., 2014	456.059	87	<0.01	0.979	0.982	0.060 (0.055, 0.066)
First- and second-order CFA in sample 2		
First-order model	245.811	74	<0.01	0.991	0.993	0.043 (0.037, 0.049)
Second-order model	245.811	74	<0.01	0.991	0.993	0.043 (0.037, 0.049)


*WLSMV, weighted least squares with mean and variance adjustment; *df*, degree of freedom; TLI, Tucker–Lewis index; CFI, comparative fit index; RMSEA, root mean square error of approximation; CI, confidence interval.*

**Table 3:** Factor correlations.

Factor	Sample 1 (*n* = 1174)			Sample 2 (*n* = 1254)		
	GDA	LS	W	GDA	LS	W
LS	0.955^∗^			0.950^∗^		
W	0.823^∗^	0.885^∗^		0.878^∗^	0.902^∗^	


*GDA, general depressive affect; LS, life satisfaction; W, withdrawal. ^∗^*p* < 0.001.*

### Second-Order CFA

As can be seen from Table 2, the second-order model had the same fit indices with the first-order model (WLSMV χ^2^ = 245.811, *df* = 74, TLI = 0.991, CFI = 0.993, RMSEA = 0.043). Standardized factor loadings for the second-order CFA were included in Table 4. The first-order factor loadings ranged from 0.552 to 0.997, showing that all items were loaded well on their latent factor. The second-order factor loadings were excellent, ranging from 0.913 to 0.987 (all >0.71).

**Table 4:** Standardized factor loadings for the second-order CFA.

Item content	GDA	LS	W
3. Your life is empty	0.879		
4. Often get bored	0.849		
6. Afraid something bad will happen	0.589		
8. Often feel helpless	0.928		
12. Feel pretty worthless	0.930		
14. Situation is hopeless	0.941		
15. Most people are better off than you	0.636		
1. Satisfied with life		0.997	
5. In good spirits		0.807	
7. Happy most of the time		0.943	
11. Wonderful to be alive now		0.620	
2. Dropped activities, interests			0.558
9. Prefer to stay at home			0.552
13. Feel full of energy			0.835
Second-order factor loadings	0.962	0.987	0.913


*GDA, general depressive affect; LS, life satisfaction; W, withdrawal.*

### Measurement Invariance Across Genders

Given that the first-order and second-order factor model had the same fit indices, we did not test the factorial invariance of the second-order model. The results showed that the three-factor model of GDS-15 is an excellent fit of the data in both males and females. Results of multigroup CFA revealed that measurement invariance across gender groups was entirely supported at the factorial structure and the strict level (see Table 5). The ΔCFIs, ΔTLIs, and ΔRMSEAs are lower than 0.01 in all models, suggesting that the gender invariance of GDS-15 has been confirmed. GDS-15 items have the same meanings across genders; that is, we can compare the latent mean differences across these groups.

**Table 5:** Goodness-of-fit indices and model comparisons for measurement invariance models.

Model	χ^2^	*df*	TLI	CFI	RMSEA (90% CI)		ΔTLI	ΔCFI		ΔRMSEA	
Females	248.289	74	0.991	0.992	0.043 (0.037, 0.049)		—		—		—
Males	279.285	74	0.988	0.990	0.049 (0.043, 0.056)		—		—		—
A	921.083	148	0.935	0.947	0.066 (0.062, 0.070)		—		—		—
B	934.327	159	0.940	0.947	0.063 (0.059, 0.067)	vs. A	0.005		0.000		–0.003
C	967.045	170	0.942	0.946	0.062 (0.058, 0.066)	vs. B	0.002		–0.001		–0.001
D	1,050.860	184	0.942	0.941	0.062 (0.059, 0.066)	vs. C	0.000		–0.005		0.000


*Model A, configural invariance; Model B, metric invariance; Model C, scalar invariance; Model D, strict invariance; *df*, degrees of freedom; TLI, Tucker–Lewis Index; CFI, comparative fit index; RMSEA, root mean square error of approximation.*

## Discussion

The 15-item Geriatric Depression Scale is a widely used questionnaire for evaluating late-life depression. This study determined the best factor structure of GDS-15 suitable for Chinese elders, and it is the first to employ second-order CFA to examine the validity of the GDS-15 total score. It is also the first study to examine the factorial invariance of the GDS-15 across gender groups among Chinese elders. The findings support that the GDS-15 is a valid instrument for screening depression and as a favorable choice in situation where economy of time is required.

Several previously reported alternative best-fit models were examined by CFA. Our CFA results revealed that the best factor structure of GDS-15 suitable for Chinese elders was the original three-factor model (i.e., general depressive affect, life satisfaction, and withdrawal). Item #10 “memory problems” was dropped from the three-factor model. The factor loadings of item 10 in other models were loaded poorly on their latent factor, suggesting that the most suitable factor structure of Chinese version GDS-15 was best explained by only 14 of the 15 items. Memory problems may be attributed to the aging process. Items (1, 5, 7, and 11) of life satisfaction were common items composing one factor (Friedman et al., 2005; Brown et al., 2007; Imai et al., 2014). Items (3, 4, 6, and 8) of the first factor were also common items composing one factor (Incalzi et al., 2003; Onishi et al., 2004). These findings indicate that the symptoms of depression are at least partly consistent across diverse geriatric populations. The best factor model of GDS-15 for Chinese elders implies the three sub-dimensions in late-life depression: general depressive affect, life satisfaction, and withdrawal. It is beneficial for us to detect and prevent late-life depression from these three aspects, which will improve the efficiency of primary care. The three factors were significantly correlated with each other both in sample 1 and in sample 2, indicating that the scale has high validity. The excellent second-order factor loadings indicated that first-order factors were adequately explained by the higher-order factor. The use of GDS-15 total score was meaningful. To the best of our knowledge, this study is the first study employing second-order factor analysis to examine the validity of the GDS-15 total score. It has significant meaning for both researchers and clinicians.

In order to compare the true differences across groups, assessment tools must be measurement invariant (Wu et al., 2012). The second purpose was to evaluate the measurement invariance of depressive symptoms across genders among Chinese elders. The three-factor structure of GDS-15 was well fitted to the data in both males and females. Multiple confirmatory factors showed that measurement invariance was supported, fully assuming different degrees of invariance. The establishment of configural invariance suggests that the number of factors and factor patterns of GDS-15 is equivalent among male and female. The determination of weak equivalence indicates that the observation items and potential factors of the scale have the same meaning across groups. Satisfying strong equivalence indicates that the cross-group difference of the observed variable mean can estimate the inter-group difference of the latent variable mean. The strict equivalence, which is the most stringent equivalent based on strong equivalence, reflects cross-group differences in latent variable variation. The results of this study confirm that GDS-15 is strictly equivalent, supporting that the GDS-15 factors have the same meaning across genders. Thus, comparisons of GDS-15 scores between men and women are meaningful. It is important that studies take measurement invariance into consideration when conducting cross-group research. Together with a recent work (He et al., 2018), our study supports the notion that the GDS (both the Long and the Short form) is a reliable, valid screening instrument for detecting depression in elderly Chinese individuals, with measurement invariance across genders. Owing to its ease of administration and short period of requirement, the GDS-15 is particularly useful in situations where the economy of time is required.

Several limitations of the present work should be acknowledged. Firstly, although all the study participants were from one of three provinces in China, they were otherwise heterogeneous in terms of gender, age, economic status, education, ethnicity, and region. These undetermined sample characteristics may exist in relation to gender differences in the GDS-15. Thus, the present results generalized to other dissimilar groups remain to be determined. Secondly, because the elderly with dementia or severe physical illness were excluded from this study, the current findings may not be applicable to these groups. Thirdly, our sample consisting of older Chinese cannot represent the worldwide population. Finally, validation of the gender invariance of this Chinese version of GDS-15 does not mean that the scale has invariance across time and culture, which should be determined in future research.

## Conclusion

In conclusion, this study found that a three-factor model fitted the underlying structure of the Chinese version of GDS-15 best. The use of GDS-15 total score is valid. In addition, the three-factor structure of GDS-15 was shown to be invariant across gender groups. Therefore, the report of significant higher GDS-15 scores of females than males reflects a true gender difference, indicating that women have more depression problems than men in aging Chinese.

## Data Availability

The datasets generated for this study are available on request to the corresponding author.

## Ethics Statement

The study was approved by the Ethics Committee of the Second Xiangya Hospital of Central South University.

## Author Contributions

All authors revised and approved the submitted version. HZ performed the initial analyses and wrote the manuscript. JH helped with collecting the data and data analysis. SY and JY supervised the study.

## Conflict of Interest Statement

The authors declare that the research was conducted in the absence of any commercial or financial relationships that could be construed as a potential conflict of interest.
